# Online communication and positive psychological capital of college students in China: the mediating role of online social support

**DOI:** 10.1186/s40359-023-01324-x

**Published:** 2023-09-15

**Authors:** Ying Jiang, Jingming Chi, Li Wang, Xiaomin Geng

**Affiliations:** 1 Graduate School of Education , Dalian University of Technology, Dalian, 116024 China; 2 State Key Laboratory of Cognitive Neuroscience and Learning and IDG/McGovern Institute for Brain Research, Beijing Normal University, Beijing, 100875 China

**Keywords:** Online communication, Online social support, Positive psychological capital, Mediating model

## Abstract

Online communication is one of the most significant factors that affect the growth and development of contemporary college students. The purpose of this study was to examine the mechanisms of online social support in the relationship between online communication and the positive psychological capital of college students in China. Drawing on social exchange theory, the research employed a sample of 1,212 Chinese college students and incorporated online social support as a mediating variable. The investigation constructed a research model using the College Students’ Online Communication Questionnaire, Online Social Support Questionnaire, and Positive Psychological Capital Questionnaire to examine the intricate relationship among online communication, online social support, and positive psychological capital. The study found that both online self-expression and online extended relationship actions positively influenced students’ positive psychological capital. However, online social participation did not demonstrate an impact on this measure. Furthermore, online social support was found to partially mediate the connection between online self-expression and positive psychological capital as well as between online extended relationships and positive psychological capital. Online social support fully mediated the relationship between online social participation and positive psychological capital.

**Supplementary Information:**

The online version contains supplementary material available at 10.1186/s40359-023-01324-x.

## Introduction

Focusing on the learning and development of college students is crucial not only for enhancing their professional skills but also for addressing their mental health. Positive psychological capital, or PsyCap, is an individual’s positive state of psychological development [1] and is considered a vital resource in positive psychology [2]. The recognized core construct of psychological capital and the first-order positive psychological resources that make up PsyCap include hope, efficacy, resilience, and optimism [3]. Previous research has shown that positive psychological capital can protect individuals from negative experiences and promote personal growth, academic achievement, and career planning for college students [4, 5]. Because positive psychological capital is a trait that can be developed and improved, identifying the factors that influence it and implementing targeted interventions can help to achieve holistic development and improve the mental well-being of college students. However, there is currently limited research that explores these factors.

Online communication specifically refers to the interpersonal communication mode in which two or more interacting entities engage in daily or nondaily communication within a virtual space based on internet technology using digital media as the means of communication [6]. Of particular interest are social networking sites (SNSs), which are defined as “websites which make it possible to form online communities and share user created content” [7]. College students are the primary users of the internet, and online communication is one of their most prevalent digital behaviors [8]. Online communication involves the interpersonal transmission of interaction signals accompanied by feedback from others, which has an inevitable impact on people’s psychological capital [9]. Research on the online communication and positive psychological capital of college students suggests that online communication fosters a conducive environment for resource sharing and exerts a beneficial influence on psychological adaptation by facilitating the acquisition of positive feedback [10]. Conversely, other studies indicate that online communication induces negative emotions, weakens emotional well-being [11], reduces self-control and satisfaction in friendship relationships [12], and can be detrimental to the development of positive psychological capital. These differing findings suggest that the underlying mechanisms by which online communication affects users’ social involvement and psychological status require further exploration. According to social exchange theory, online communication involves interpersonal interactions accompanied by a process of resource exchange [13]. Online communication can increase social capital [6] and individuals’ perceptions of social support [14] and influence the quality of friendship by allowing people to access social support through online channels [15]. The perception of social support in a network leads to a more positive psychological state for individuals [16]. Therefore, online social support, as a form of positive feedback from others [17], may play an important mediating role.

College students face significant new pressures and challenges due to the increasing demands of networks [6]. In the midst of online technological advances, it is necessary to consider the developmental influences these new technologies have on college students [18]. Understanding the effects of online communication on college students’ positive psychological capital is important to correct their normative perceptions of online communication and promote college students’ psychological health in the online era. Furthermore, current research on online communication lacks a theoretical perspective to delineate the dimensions of online communication, and it inadequately incorporates the concept of online social support from the perspective of social exchange. Based on this, this research classifies online communication according to communicative action theory and reveals the mechanism by which online communication influences positive psychological capital through social exchange theory.

## Theoretical analysis and research hypothesis

### Theoretical analysis

Online communication refers to interpersonal interactions between people by means of text, voice, or video based on network terminal equipment and internet technology [19]. Online communication provides more forms of expression for interaction, such as retweeting, posting, and replying [20–22]. These unique online communication actions contain deeper internal logic, which plays an important role in the establishment and maintenance of interpersonal relationships. According to Habermas’ theory, communicative action involves the association of the subjective world, the objective world, and the social world at the same time [23]. Thus, this research categorizes online communication into three types based on the emphasis on three aspects of the world: online self-representation action, online extended relationship action, and online social participation action. Online self-representation action is the action of exploring one’s own online world to present a certain social image to others and to seek recognition, such as posting pictures, text, and video. Online extended relationship action is the action of using online communication functions to establish, develop, and maintain close relationships over the internet, such as sharing and following friends’ updates. Online social participation action is the action of expressing one’s thoughts, ideas, and values on the internet, such as comments on hot topics.

In the 1960s, social exchange theory (SET) was established in the United States and began to spread in Western Europe [24]. It aimed to account for interactions between individuals in terms of exchange relationships [25]. The basic viewpoint is that social interaction is formed based on value exchange, and any social interaction depends on the value exchange of different things [26]. According to social exchange theory, online communication is an interpersonal interaction and a symbolic transfer process in which two parties in a relationship provide resources to each other or negotiate an exchange of resources. Social activity arises when there are resources that are mutually needed by both parties in society. These resources include material rewards such as money, status, and services as well as spiritual aspects such as care and information [26]. The physical or psychological price we pay can increase the possibility of obtaining resources and thus increase the positive psychological resources of individuals [27]. The significance of social exchange theory lies in the view of online interactions as a process of comparing benefits and costs. The time and effort individuals expend during online communication can be transformed into tangible gains (such as information and material resources) and psychological rewards (such as gaining recognition and social approval). Online communication possesses unique mechanisms to trigger social exchanges in which the avenues for gaining benefits are more convenient and the costs are lower. These motivations provide almost unrestricted access to interactive symbols, tools, and means. Furthermore, online communication emphasizes individual agency, allowing individuals to decide whether to engage in or sustain online communication. Individuals can also proactively terminate online communication if the benefits fall below the costs.

According to the process of resource exchange, there are two types of effects between online communication and positive psychological capital. Online social support, as a type of positive feedback from others, may be an important mediating variable for online communication to influence college students’ positive psychological capital. Therefore, this paper analyzed the process by which online communication affects positive psychological capital from the perspective of social exchange theory. This process comprises self-interaction and interaction with others, which can have both direct and indirect effects. Online social support, as a form of positive feedback from others, may play an important mediating role in the relationship between online communication and positive psychological capital among college students in China.

The social characteristics of the interpersonal relationship school suggest that obtaining social support provides important social security for the continuous development of “social individuals” [28]. In other words, regardless of whether it is real-life communication or online communication, social support is the driving force for communication. However, online social support is not one-way care and mutual assistance, and in most cases, it is a result of social exchange. Generally, the positive feedback obtained by individuals after online communication is a collection of resources. In the process of social exchange, there are three exchange results: the gain of resources, the loss of resources, and the balance of income and expenditure of resources [29]. Social exchange theory emphasizes that both parties must follow the principle of reciprocity; that is, one party must repay the other party for resources obtained, which involves the consumption of individual resources and can increase individual resource gains. Only the loss of resources will have a negative impact on online social support [30]. Therefore, achieving smooth communication requires the achievement of a state of “balance of income and expenditure”. If social support is obtained in the process instead of the loss of personal resources, it can promote the formation and development of positive psychological capital.

### Relationship between online communication and positive psychological capital

People participate in online communication to obtain one type of benefit that involves both an increase in actual resources and the pleasurable nature of the psychological experience. This advantage emerges from the process of interaction between two or more people, which is not the same as watching news or videos over the internet. Online communication does not have the necessity of real communication, so it can break the pressures and constraints of real communication. It places more emphasis on individual initiative, and individuals can decide on their own whether to engage in online communication activities and can anticipate the results.

Online communication is actively selected for performance driven by individuals’ internal motivation and thinking, which can enhance interactions with friends and promote positive effects, similar to subjective well-being [31]. Due to the absence of visual cues in online communication, individuals achieve effective exchanges of information and emotional communication between two parties through higher levels of self-expression. The online environment also provides a new field for people to observe others’ reactions and receive recognition from others, and self-expressive action has a positive impact on both the individual self and psychological adaptation and can help people obtain positive feedback [10]. Online extended relationships can broaden the scope of friendships and create more communication opportunities. Making friends and maintaining relationships to meet emotional needs can promote the accumulation of positive psychological capital among college students [32]. In addition, online media amplifies individual expression in online communities but weakens the consideration of identity and character in anonymity. Viewers and short video creators share and discuss social issues, giving participants new ways of thinking and diverse choices and promoting individuals’ perception of positive psychological capital [33]. Therefore, the following hypothesis is proposed.

H1: Online communication has a positive effect on positive psychological capital.

### Relationship between online communication and online social support

According to social exchange theory, human interaction is a process of resource exchange. The purpose of exchange is to obtain “rewards”, and various types of exchanges are equivalent to “rewards”. Driven by resource interests, members of society engage in social interaction with the aim of satisfying their own needs or obtaining “rewards” in the process of interaction.

College students initiate online interaction to signal that they want to achieve a certain kind of interaction. The main resources exchanged are nonmaterial resources such as caring and information, so online communication helps people obtain online social support [34]. First, compared with offline self-expressive action, online self-expressive action enables individuals to obtain praise and exchange information [35]. Second, the number of friends contacted online is much larger than the number of offline interpersonal interactions, and this large number of friends can provide a large amount of information and emotional support [36]. Finally, the internet provides a platform for college students to discuss various topics, which increases their enthusiasm for engaging in conversations about current events and creates a community for exchanging both information and emotions. This platform can be helpful for college students seeking valuable opinions, information, and emotional support [37]. Therefore, the following hypothesis is proposed.

H2: Online communication has a positive effect on online social support.

### Relationship between online social support and positive psychological capital

Positive psychology posits that every individual possesses both negative and positive forces of resistance that can override or complement each other. The determining factor is which force an individual continually energizes to create a suitable psychological environment for survival. Consequently, online social support provided by communication platforms has become an important means for college students to obtain positive energy in this dynamic environment.

The creation of the internet has widened the range of interpersonal interactions and increased online social support, which enables people to obtain social resources that can influence their personal development, thus affecting the formation of positive psychological capital [38]. Although some studies have concluded that online social support is positively correlated with internet addiction [39], most researchers treat online social support as a social resource that contributes to the survival and development of individuals. It is believed that there is a direct relationship between online social support and psychological health [40], with a higher level of online social support corresponding to a higher level of psychological health [41]. Fragmentation can disperse knowledge into massive, fragmented and diversified information that caters to the individual needs of college students who have convenient access to social support [42]. Therefore, the following hypothesis is proposed.

H3: Online social support has a positive effect on positive psychological capital.

### The mediating role of online social support

With regard to social support, the more resources individuals have available to meet their needs, the better they will be able to cope with various challenges from the environment [43]. Adequate social support can facilitate the development of positive psychological traits in individuals. Therefore, the relative resources between interacting parties are the inherent behavioral logic of social interactions, and the information and emotional resources obtained in the process of online communication can produce online social support [44].

Online social support is characteristic of the new generation, and it has become an important way for college students to obtain social support. In the process of online communication, stressful events can arise due to loopholes in the interaction system, the vulgarization of interaction content, the differentiation of interaction objects, and miscommunication of words. Online social support can help individuals cope with stressful events [45]. First, online social support can serve as a moderator between potentially stressful events and an individual’s perception of stress. This can attenuate the perception of these events and reduce the level of stress they cause. Second, in relation to perceived stress, online social support can provide solutions to difficulties and emotional comfort to mitigate the adverse effects of the stressful event. This is because social support, such as acceptance, caring, trust, and encouragement, delivered during online communication can make the receiver feel cared for, respected, and valued. This can reduce the individual’s sense of loneliness and provide physical and psychological satisfaction, which benefits both parties physically and mentally. Therefore, the following hypothesis is proposed.

H4: Online social support mediates the relationship between online communication and positive psychological capital.

## Materials and methods

### Participants and procedure

The questionnaire collection process used the Chinese software “Questionnaire Star” (https://www.wjx.cn/login.aspx) to distribute and collect questionnaires online. According to the proportion of Chinese university students, the selection of universities included one “Double First-Class” construction university, four regular undergraduate colleges, and five schools from different categories: engineering and science, economics and finance, medical, normal and teacher training, and agriculture and forestry. Considering that students from economically developed provinces are more likely to use online communication, we sampled universities from Liaoning, Shandong, Jiangsu, and Zhejiang, all of which are located in open coastal cities in eastern China. We employed a simple random sampling method to select two classes, utilizing the spare time during common courses for large-screen projection. We also distributed questionnaires for students to fill out in class groups, study groups, communication groups, and entertainment groups. A total of 1212 questionnaires were initially collected. After filtering out those with obvious errors, 1065 questionnaires with complete and valid data were included for analysis. Of the participants, 614 (57.7%) were male, 451 (42.3%) were female, 375 (35.2%) were from rural areas, and 690 (64.8%) were from urban regions.

### Measures

#### Online communication

The theoretical framework of online communication was developed from Habermas’ theory and contains three dimensions: online self-expressive action, online extended relationship action, and online social participation action. The questionnaire that was developed, the “Internet Interaction Questionnaire for University Students” [46], was modified to compile the items. The Internet Interaction Questionnaire for University Students had the following results: Cronbach’s alpha = 0.90, retest reliability = 0.88,χ^2^/df = 3.59, RMSEA = 0.094, CFI = 0.89, NNFI = 0.87, and IFI = 0.89 [46]. After item analysis and exploratory factor analysis of the initial questionnaire, the final 21 question items (appendix) were formed and scored using a 5-point Likert scale. Summed scores were analyzed, with higher scores indicating greater online communication. The results showed that the scale had good reliability and validity [47].

Online self-expressive action: Cronbach’s alpha = 0.809, χ^2^/df = 1.087, RMSEA = 0.044, IFI = 0.989, TLI = 0.980, NFI = 0.984.

Online extended relationship action: Cronbach’s alpha = 0.854,χ^2^/df = 2.568, RMSEA = 0.060, IFI = 0.935, TLI = 0.913, NFI = 0.928.

Online social participation action: Cronbach’s alpha = 0.814, χ^2^/df = 2.153, RMSEA = 0.058, IFI = 0.982, TLI = 0.955, NFI = 0.980.

#### Online social support

Based on the research results of the “Online Social Support Questionnaire for University Students” [48], we divided online social support into two dimensions, online information support and online emotional support, and adapted them on this basis. The questionnaire consisted of 8 items (e.g., “Through the internet, I get some learning materials and learning experiences from others”, “When I feel lonely, I talk to people through the internet”) and was scored on a 5-point Likert scale. The summed scores were analyzed, with higher scores indicating greater online social support. The results showed that the scale had good reliability and validity (Cronbach’s alpha = 0.884, χ^2^/df = 2.711, RMSEA = 0.067, IFI = 0.982, TLI = 0.974, NFI = 0.978).

#### Positive psychological capital

The “Positive Psychological Capital Questionnaire” developed by Zhang [49] was used to assess the level of positive psychological capital of college students. The questionnaire consists of 26 items, including four dimensions of self-efficacy, optimism, resilience and hope, and is scored on a 7-point scale, ranging from “not at all” to “completely”, with higher scores indicating higher storage of positive psychological capital. The results showed that the scale had good reliability (Cronbach’s alpha = 0.917).

### Data analysis

We first performed descriptive statistical analysis, independent-sample t tests and Pearson correlation analysis among the study variables. To examine the mediation effects, we utilized the PROCESS macro in SPSS 24.0 [50]. A simple mediation model (Model 4) was employed to assess Hypothesis 4. All continuous variables were standardized before conducting the regression analyses. The significance of the findings was determined using the bias-corrected percentile bootstrap method with 5,000 resamples and a 95% confidence interval (CI). We considered the model statistically significant when the 95% CI excluded zero.

## Results

### Basic conditions of college students’ online communication

#### Descriptive statistical analysis

Regarding the time spent on online communication, 47% of college students reported spending between 2 and 4 h engaging in such activities, which is considered reasonable. Furthermore, 88% of college students reported spending less than 6 h on online communication. In terms of their perception of online communication, the majority of college students acknowledged its significance in interpersonal communication, with 86% considering it an integral part of interpersonal communication. Additionally, approximately 86% of college students believed that online communication affected their offline communication, with 42.4% perceiving a more positive impact, 34.5% perceiving both positive and negative impacts equally, and only 2.4% considering the negative impact more significant. However, approximately 20% of college students viewed online communication and offline communication as independent of each other.

#### Difference test

There were differences (P < 0.05) in college students’ online communication in terms of gender, number of siblings, and place of birth. The results are shown in Table 1.

**Table 1:** Results of the test of variance for online communication behavior

Dimension	Gender	M ± SD	No siblings	M ± SD	Place of birth	M ± SD
Online self-expressive action	Male	3.468 ± 0.774	Yes	3.506 ± 0.709	Rural	3.297 ± 0.754
	Female	3.381 ± 0.630	No	3.327 ± 0.718	Urban	3.504 ± 0.687
t		2.011^*^		4.042^***^		-4.520^***^
Online extended relationship action	Male	3.447 ± 0.798	Yes	3.507 ± 0.778	Rural	3.266 ± 0.775
	Female	3.403 ± 0.705	No	3.319 ± 0.721	Urban	3.517 ± 0.738
t		1.952^*^		4.014^***^		-5.271^***^
Online social participation action	Male	2.812 ± 0.984	Yes	2.780 ± 0.981	Rural	2.558 ± 0.952
	Female	2.557 ± 0.868	No	2.599 ± 0.880	Urban	2.784 ± 0.931

### Common method biases test

The self-reported data in this study may have potential biases, so Harman’s single-factor test was conducted to check for common methodological biases before the data analysis [51]. The results of the exploratory factor analysis showed that all variable eigenvalues were greater than 1 and the first common factor accounted for 29.316% of the variance, which is less than 40%, indicating that there were no significant common biases in the study [52].

### Descriptive and correlation analysis

According to the correlation analysis, college students’ online self-expressive action, online extended relationship action, and online social participation action were positively correlated with online social support and positive psychological capital. Online social support was also positively correlated with positive psychological capital, which provides support for further exploration of the relationship among variables and testing of the mediating effect of online social support. The means, standard deviations and correlation coefficients of the variables in the study are shown in Table 2.

**Table 2:** Means, standard deviations and correlation coefficients among variables

	mean	standard deviation	online self-expressive action	online extended relationship action	online social participation action	online social support
online self-expressive action	3.431	0.718				
online extended relationship action	3.429	0.760	0.532**			
online social participation action	2.704	0.944	0.441**	0.590**		
online social support	3.739	0.768	0.394**	0.613**	0.434**	
positive psychological capital	3.788	0.724	0.349**	0.469**	0.385**	0.486**

### Model validation analysis

#### Path analysis

**Fig. 1:**
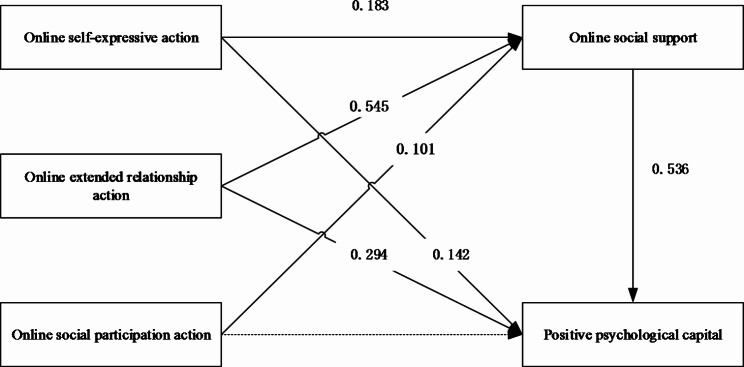
Analysis model of online communication on positive psychological capital path

Regression analysis was used to verify the process paths of online self-expressive action, online extended relationship action, and online social participation action with positive psychological capital and with online social support. The results of this study showed that online self-expressive action and online extended relationship action had a significant positive effect on college students’ positive psychological capital (β = 0.142, p < 0.01; β = 0.294, p < 0.01), while online social participation action had no effect on college students’ positive psychological capital. Hypothesis H1 was partially verified. Online self-expressive action, online extended relationship action, and online social participation action significantly predicted online social support (β = 0.183, p < 0.01; β = 0.545, p < 0.01; β = 0.101, p < 0.01). Hypothesis H2 was verified. Online social support significantly predicted positive psychological capital (β = 0.536, p < 0.01). Hypothesis H3 was verified. The results are shown in Fig. 1.

#### Mediation effect test

To test the mediation effect of online social support between online communication and positive psychological capital, a simple mediation model of online communication, online social support, and positive psychological capital was constructed. According to the mediation test method, Model 4 in the PROCESS plug-in written by Hayes was used to explore the mediation effect of online social support between online communication and positive psychological capital [50]. The specific results are shown in Table 3.

**Table 3:** Mediated path effect value 95% confidence interval Boot SE

Effect	效应值	Boots 95% CI		Boot SE
		LLCI	ULCI	
Online self-expressive action → Online social support → Positive psychological capital	0.118	0.003	0.053	0.013
Online extended relationship action → Online social support → Positive psychological capital	0.144	0.135	0.227	0.024
Online social participation action → Online social support → Positive psychological capital	0.001	0.009	0.056	0.012

The indirect effect value of online self-expressive action on positive psychological capital was 0.118 with a point estimate 95% confidence interval of [0.003, 0.053], indicating a significant mediating effect of online social support in the influence of online self-expressive action on the positive psychological capital of college students with a mediating effect percentage of 16.947%. The indirect effect of online extended relationship action on positive psychological capital was 0.144 and the point estimate 95% confidence interval was [0.135, 0.227], indicating that the mediating effect of online social support in online extended relationship action on the positive psychological capital of college students was significant, accounting for 50.961%. The indirect effect value of online social participation action on positive psychological capital was 0.001 and the point estimate 95% confidence interval was [0.009, 0.056], indicating that the mediating effect of online social support in online social participation action influencing the positive psychological capital of college students was significant. According to the method for judging the mediating effect [53], the direct effect of online social participation on positive psychological capital was not significant. Therefore, only the mediating effect of online social support was observed. It was found that online social support played a full mediating role in online communication from a mediation effect perspective. Hypothesis H4 was verified.

## Discussion

We investigated the mediating effects of online social support on the relationship between online communication and positive psychological capital. Drawing on Habermas’s theory of communicative action, the dimensions of online communication were divided into online self-expression action, online extended relationship action, and online social participation action. Online social support was introduced as a mediating variable to reveal the effects of different online communication actions on college students’ positive psychological capital, and the main findings and insights are as follows.

### Basic situation of online communication among college students in China

Regarding the time spent on online communication, it is noteworthy that 88% of Chinese college students spent less than 6 h, indicating that their engagement in online communication activities was within a reasonable time frame. Furthermore, the majority of college students recognized the importance of online interactions in fostering and maintaining interpersonal relationships, with 86% considering online interactions a vital component of these relationships. Interestingly, the majority of college students held a positive perception of online communication, with only 2.4% perceiving it to have a negative influence. This finding highlights that online communication is widely accepted among college students and is not typically viewed as having a detrimental impact on users. Overall, college students currently spend a moderate amount of time engaging in online communication activities, and the majority of them recognize the benefits of these interactions.

Regarding the current status of online communication, research shows that male college students engaged in more online communication than their female counterparts, which is consistent with findings from other countries [54, 55]. Additionally, only children tend to engage in more online communication than non-only children, potentially because online communication compensates for the lack of peer interaction. This aligns with the compensation hypothesis, which proposes that online communication provides a new means of interaction for only children to alleviate loneliness and achieve greater satisfaction compared to children with siblings. On the other hand, urban college students generally engage in more online communication than their rural peers, likely due to differences in economic status that enable urban students to access and become accustomed to online communication at an earlier age, which provides them with a broader range of social connections and communication opportunities.

### The relationship between online communication and positive psychological capital

Online communication possesses similar interaction properties as face-to-face communication and may positively impact individuals’ positive psychological capital during interpersonal communication. Online communication not only makes communication faster and more convenient but also enables more personal initiative, allowing for greater possibilities in self-expression, the maintenance of relationships, and social participation. However, the effects of online communication on college students’ positive psychological capital vary depending on the type of communication action. Online self-expression and extended relationship action have a positive impact on positive psychological capital, while online social participation has no direct effect. In other words, online communication does not necessarily have a positive effect on individuals’ psychological development. Positive psychological capital, as a psychological resource, can only be obtained by paying attention to the psychological aspects of individuals.

College students are at an age when they are about to enter society and, as such, are often concerned with their appearance and body shape, which may create a strong need for self-expression. Self-expressive actions in network interactions not only showcase oneself to others but also bring personal pleasure. Furthermore, online communication can expand one’s social circle, provide positive feedback, and foster recognition from others, all of which promote positive psychological capital. However, it is crucial to acknowledge that the current online environment is mixed, and individuals of various levels are present online. This often leads to the dissemination of incorrect ideologies or negative influences that can offset the positive effects of online social participation, rendering it ineffective in promoting positive psychological capital.

### Mediating role of online social support

This study also found that online social support mediates the relationship between online communication and positive psychological capital. The more frequent the action of online communication, the greater the possibility of gaining online social support and the wider the range. Online communication makes the path to obtain social support easier and the ways to harvest social support richer for college students. College students’ online social support may directly influence health-related factors, reduce or prevent psychological problems, relieve life and study stress, enhance positive psychological capital, and contribute to the formation of positive psychological capital among college students. Although college students’ online social participation cannot directly influence positive psychological capital, students can positively influence their positive psychological capital by obtaining online social support. In other words, online social participation action can positively influence social resources and have a positive pull effect on psychological resources through positive social resources, so online social participation mainly acts indirectly on positive psychological capital by enhancing online social support.

## Conclusion and implications

The study proposed two major hypotheses. The first is that online communication can influence college students’ positive psychological capital. The second postulates that online social support mediates the relationship between online communication and positive psychological capital. The results indicate that different types of online communication action have varying effects on positive psychological capital. Specifically, online self-expression action and online extended relationship action have direct and positive impacts on positive psychological capital, while online social participation has no effect. Analyzing the mediating effect of online social support, the results suggest that online social support partially mediates the relationship between online self-expression action and positive psychological capital as well as between online extended relationship action and positive psychological capital. However, online social support fully mediates the relationship between online social participation and positive psychological capital. These findings suggest that online social participation and positive psychological capital are not simply directly related but instead are influenced by online social support.

These findings confirm the positive impact of online communication on college students’ positive psychological capital. It is evident that online communication represents a form of “controlled social interaction”, where successful engagement depends primarily on the user’s initiative. Habermas’ theory of communicative action emphasizes the importance of sincerity, truthfulness, and correctness for effective action. Thus, online communication characterized by sincere, truthful, and correct interactions is essential for promoting positive outcomes. Online social support has been shown to reduce or prevent psychological problems among college students and to contribute to the formation of positive psychological capital. However, it is crucial to possess the ability to discern whether information obtained from online strangers is accurate and genuine and whether the respect and satisfaction gained are also sincere and effective. In accordance with social exchange theory, obtaining desired resources is critical to successful online communication. Social interactions require people to seek mutual interests, and smooth interactions can be achieved only through reciprocity. College students should focus on the value of interaction in online communication to pursue and maintain meaningful communication.

## Limitations and future research directions

There are limitations in this empirical study due to factors such as our own capabilities and research conditions. The following issues emerged in the process. First, the use of only cross-sectional data limited causal inferences. Future research could consider longitudinal tracking. Second, this study employed a convenience sampling method and selected university students from four provinces and five schools as the sample. In the future, it would be beneficial to broaden the scope of the study. Finally, the data collection process for the study took place after the COVID-19 pandemic. Although this study made efforts to exclude the impact of the pandemic on human psychology as much as possible through theoretical construction and literature collection, it is undeniable that the influence of specific periods cannot be completely eliminated. We encourage readers to understand our research context and encourage further studies to validate and supplement our findings to ensure the robustness and replicability of the results.

The results of this research suggest that the online communication of college students in China is influenced by various antecedents, including external environmental and individual factors. Future research could explore the influence of family background and interaction motivation as potential antecedent factors of online communication action. Furthermore, positive psychological capital comprises multiple dimensions and qualities that may be differentially impacted by online communication action. Further research could investigate the impact of different online communication actions on various dimensions and traits of positive psychological capital to provide a deeper understanding.

### Electronic supplementary material

Below is the link to the electronic supplementary material.

Supplementary Material 1

## Data Availability

The datasets generated and analyzed in the current study are available from the corresponding author upon reasonable request.
