# Supplementary material for: Online communication and positive psychological capital of college students in China: the mediating role of online social support
Source: BMC Psychol. 2023 Sep 15;11:277. doi: 10.1186/s40359-023-01324-x (PMC10504745; doi:10.1186/s40359-023-01324-x)
Supplement: Supplementary file 1 — Supplementary Material 1 [file 40359_2023_1324_MOESM1_ESM.docx]

**Supplementary File**

**Appendix**

Online Communication Questionnaire for College Students

| Dimension | Subject |
| --- | --- |
| Online self-expressive action | *B_1_*：I can honestly record my negative emotions (e.g. sadness, anxiety or anger) |
|  | *B_2_*：When I update my status, I don't mind writing about something bad that happened to me |
|  | *B_3_*：When updating my status, I often selectively express positive feelings |
|  | *B_4_*：When posting, I just want to show the best of myself, no matter what the reality is. |
|  | *B_5_*：Regardless of my true feelings, I often use happy words or emoticons when sending messages |
|  | *B_6_*：In networking, I will choose to show my popular side and hide socially unpopular traits |
| Online extended relationship action | *B_7_*：I usually take the initiative to join different types of WeChat groups, QQ groups and other groups |
|  | *B_8_*：When I want to meet more people or learn a new skill, I often search the web to add friends! |
|  | *B_9_*：I'm always active in networking groups and making friends. |
|  | *B_10_*：On the web, I often communicate with close friends |
|  | *B_11_*：When I come across an interesting video or article, I often share it with good friends |
|  | *B_12_*：I often tell my buddies about the knowledge I've gained online |
|  | *B_13_*：In social networking sites, I often like my friends' updates |
|  | *B_14_*：I'm always following my friends. |
|  | *B_15_*：I often comment under my friends' updates |
|  | *B_16_*：When I know that a friend needs to be retweeted and publicized, I will retweet and publicize for the friend |
| Online social participation action | *B_17_*：When comments in the internet don't match what I think is the truth, I often point out the problem |
|  | *B_18_：*I often comment when faced with hot issues on the internet |
|  | *B_19_*：I often create my own articles, videos, pictures, music, etc. in the web |
|  | *B_20_*：I'm running or writing for a public website. |
|  | *B_21_*：I often post messages expressing my thoughts in the internet |
